# Comparative genomic analysis reveals the environmental impacts on two Arcticibacter strains including sixteen Sphingobacteriaceae species

**DOI:** 10.1038/s41598-017-02191-4

**Published:** 2017-05-17

**Authors:** Liang Shen, Yongqin Liu, Baiqing Xu, Ninglian Wang, Huabiao Zhao, Xiaobo Liu, Fei Liu

**Affiliations:** 1 Key Laboratory of Tibetan Environment Changes and Land Surface Processes, Institute of Tibetan Plateau Research, Chinese Academy of Sciences, Beijing, 100085 China; 2 CAS Center for Excellence in Tibetan Plateau Earth Sciences, Beijing, 100085 China; 3 University of Chinese Academy of Sciences, Colledge of Resources and Environment, Beijing, 100049 China; 4 College of Urban and Environmental Science, Northwest University, Xian, 710069 China; 5 Institute of Microbiology, Chinese Academy of Sciences, Beijing, 100101 China

## Abstract

How the genomic diversity of species is driven by geographical isolation and environmental factors are not well understood for cold environments. Here, the environmental stress responses of two phylogenetically close *Arcticibacter* strains, *A. eurypsychrophilus* MJ9-5 and *A. svalbardensis* MN12-7, isolated from a Tibetan Plateau glacier and Svalbard soil, were analyzed. The comparative genomic analysis was performed with sixteen other related Sphingobacteriaceae species. Analyses of the relationships between growth temperature and genome composition, cold and heat shock genes showed that genomic adaption characteristics were more obvious when the strains were grouped by their upper limit in growth temperature, rather than by their minimal or optimal growth temperatures for Sphingobacteriaceae species. The very divergent genetic distance of genome fractions assigned to the functions of ‘secondary metabolism’, ‘dormancy and sporulation’ and ‘metabolism of aromatic compounds’ indicated the heterogeneous evolution of genes under different environmental pressures of the Sphingobacteriaceae species. The greatest differences between strains MJ9-5 and MN12-7 occurred in the genes devoted to the CRISPRs, osmotic adaption and metabolism of monosaccharides, nitrogen and aromatic compounds. These distinctions corresponded to two different environmental pressures, salinity and nutritional level, in the glacier ice and Svalbard soil environments.

## Introduction

The adaption mechanisms of organisms in cold aquatic, glacier and permafrost environments have received wide attention^1–3^. Culture dependent and independent studies revealed that the cryosphere, the coldest biome, harbors a considerable number of microbes, which are well adapted to the low temperatures^4, 5^. With the rapid development of sequencing techniques and other “omic” techniques, it has been possible to investigate cold adaption mechanisms at the genetic, proteomic and metabolomic level on a large scale^6–9^. A genomic study of *Colwellia psychrerythraea* 34H, isolated from Arctic marine sediments, revealed that cold adaption in the species is most likely conferred by a collection of synergistic changes across the whole genome’s content and amino acid composition, not by a unique set of genes^10^. Genomic analysis of an extreme psychrophile, *Psychromonas ingrahamii*, which grows exponentially at −12 °C and was isolated from Arctic sea ice, showed in detail how the proteins had changed to adapt to the low temperatures^11^. Allen *et al*.^3^ suggested that genome plasticity played an important role in the adaptation of strain *Methanococcoides burtonii* DSM 6242, which moved from a marine to an Antarctic lake environment^3^. A strain of *Planococcus halocryophilus*, isolated from high Arctic permafrost, is able to grow and divide at −15 °C and analysis of the genome revealed that cold and osmotic-specific adaptive mechanisms were present, along with an increased flexibility of proteins in the organism^1^. More recently, genomic and phenotypic analyses of *Arthrobacter* from Antarctic soils revealed isolates that contained several features, such as genes primarily assigned to sigma factors, the carotenoid biosynthesis and genes induced by cold-shock, oxidative and osmotic stresses, which may be beneficial for growth and survival in the Antarctic soil environment^12^.

Whole genome studies extend knowledge and understanding, characterizing the ways that microorganisms’ genomes have adapted to different habitats. However, few physiological studies are conducted alongside genomic analyses; in some cases a general genomic adaption profile has been applied to all cold tolerant bacteria. There has also been a lack of phylogenetic analyses, compared with numerical analyses of function assignable genes^11–13^. Furthermore, studies of inland glacier bacteria, an important part of the cryosphere biome, are rarely conducted^5, 14^.

The bacterial genus *Arcticibacter* is a newly described Bacteroidetes cluster in the well-studied order, Sphingobacteriales, the organisms of which are renowned for their ability to produce extracellular polymeric substances and degrade recalcitrant molecules^15–17^. When first established in 2013, the genus *Arcticibacter* contained one species, *A. svalbardensis*, which was isolated from an Arctic soil. The present study compared the physiological and genomic characteristics of two strains of *Arcticibacter*: *A. eurypsychrophilus* MJ9-5 (hereafter referred to as strain MJ9-5) and *A. svalbardensis* MN12-7 (hereafter referred to as strain MN12-7); these were isolated from a Tibetan Plateau glacier and an Arctic Svalbard soil, respectively^15, 18^. To the best of our knowledge, the Arctic Svalbard soil and the Tibetan Plateau are the only known sources of the genus *Arcticibacter* to date. Because of its unique distribution, *Arcticibacter* could be used as a model microbe for the study of genetic evolution during adaption to local environments. Strain MJ9-5 was isolated from an ice core taken from the Muji glacier on the Tibet Plateau. The genus *Arcticibacter* made up 36% of the cultivable bacteria from the ice core, followed by *Flavobacterium* (21%) and *Polaromonas* (15%). *Kocuria*, *Sphingomonas*, *Mycetocola* and *Arthrobacter* made up the remaining 28%. However, their adaption strategies are not yet well understood. Muji glacier is located in the northwest of the Tibetan Plateau (73.75°E, 39.18°N). The annual average air temperature of Muji glacier was ~ −11.5 °C. The average concentration of main cationic and anion were: Na^+^, 221.06 ng/g; NH_4_^+^, 21.89 ng/g; K^+^, 229.28 ng/g; Mg^2+^, 357.51 ng/g; Ca^2+^, 6811.75 ng/g; Cl^−^, 277.26 ng/g; NO^3−^, 532.86 ng/g and SO_4_^2−^, 2588.30 ng/g. Dissolved organic carbon was ~1.73 mg/L, and the total nitrogen was ~0.17 mg/L. The main ionic concentrations of Muji glacier was less than one fifth of those in soil of Svalbard^19^.

The aim of the present study was to clarify genomic adaptions to ice habitats alongside physiological characteristics, and to identify the genomic factors that have shaped the unique distribution of *Arcticibacter*. Genomic comparisons among closely related species from different environments can reveal the degree of genomic evolution and help to understand how environmental factors shape genomic differentiation. Thus, 16 other Sphingobacteriales strains, for which genomic data were available at the time of analysis, were also used for comparison.

## Materials and Methods

The genomic DNA of strain MJ9-5 was extracted using the method previously described by Marmur *et al*.^20^, from cells grown on R2A for 3 days at 20 °C. The purity of genomic DNA was assessed with a NanoDrop (2000c, Thermo Scientific, USA) and had an OD 260:280 ratio of 1.8–2.0. The DNA was stored in TE buffer (pH 8.0) for genome sequencing.

The genome of strain MJ9-5 was sequenced using an Illumina Hiseq 2000. Short reads were assembled using SOAPdenovo (K-mer = 31), a genome assembler developed specifically for next-generation short-read sequences^21^. As the algorithm is sensitive to sequencing errors, low-quality reads were filtered, and high-quality reads were used for *de novo* assembly. We filtered low quality reads (Phred score < 20), and then removed sequence reads shorter than 25 bp. The SOAP GapCloser was then used to close gaps after assembly. The Whole Genome Shotgun sequences were deposited at DDBJ/ENA/GenBank under the accession number MDFN00000000. The version described in this paper is version MDFN01000000.

Reference genomes of strain MN12-7 and the 16 other Sphingobacteriales strains were downloaded from the National Center for Biotechnology Information (NCBI). The completeness of genomes was calculated using CheckM^22^. To remove potential differences introduced through different annotation methods, all the genomes analyzed were annotated simultaneously in the present study. Functions were assigned through comparisons against multiple databases, including NR (non-redundant) protein databases, RAST (Rapid Annotation using Subsystem Technology) and the SEED project^23, 24^. Each predicted gene was assigned a unique identifier, prefixed with the appropriate abbreviation, for example, ‘Aeur’ for strain MJ9-5 (*A. eurypsychrophilus*) and ‘Asva’ for strain MN12-7 (*A. svalbardensis*). 16S rRNA genes for phylogenetic analysis were generated from the genomes using RNAmmer (v.1.2)^25^.

An all-versus-all search was performed with BLAST + 2.2.28, with an E-value cutoff of 1e-5. The argument ‘-negative_gilist’ was used when processing with the references genomes already in the NR database. Genes without orthologs were considered to be specific genes. Genes that had no hit in the order Sphingobacteriales were considered as horizontal gene transfer (HGT) genes and were identified using the method described by Qin *et al*.^2^.

Average nucleotide identity (ANI) was calculated with the PERL script ‘ANI.pl’ (https://github.com/chjp/ANI/blob/master/ANI.pl). Clustered regularly interspaced short palindromic repeats (CRISPRs) were found with CRISPR-finder^26^. Codon usage, amino acid composition and protein comparisons between genomes were carried out with the PERL scripts ‘CodonAaUsage.pl’, ‘aminoacidUsage.pl’, and the programs ‘matrix_createConfig’ and ‘matrix’^27^. Heat maps were produced with R^28^. Multiple alignments were performed using ClustalW, and topology trees of the 16S rRNA genes and functional genes were constructed using MEGA v5.0, by bootstrap (1000 iterations)^29^.

The genomes were split into subcategories, and calculations and text processing, such as extracting sequences from genome files and parsing BLAST outputs, were performed using custom-made PERL scripts, which are available from the authors on request.

The growth of strains MJ9-5 and MN12-7 in the presence of 0–8% NaCl (w/v) at intervals of 0.2% was investigated in R2A broth. Growth at various temperatures (−1 °C to 40 °C) was also measured in R2A broth in parallel. For the −1 °C and 0 °C temperature treatments, the temperatures were maintained with ice–water mixtures and by controlling the ambient temperatures at 0 °C and 4 °C, respectively. The R2A broth remained liquid at −1 °C. Other temperature treatments were sustained using a constant-temperature incubator. Assimilation of carbon source was determined by API 20NE and strips (bioMe´rieux) according to manufacturer’s instructions. Hydrolysis of starch and glycogen was performed according to Smibert *et al*.^30^.

To monitor growth, absorbance was measured at 600 nm on a Microplate Reader (MD SpectraMax M5). Uninoculated tubes served as control blanks and allowed verification of no contamination. The morphology of cells harvested from the 15 °C and 20 °C treatments was examined by transmission electron microscopy (JEM-1230, JEOL).

## Results

### Phenotypic characteristics

**Figure 1:**
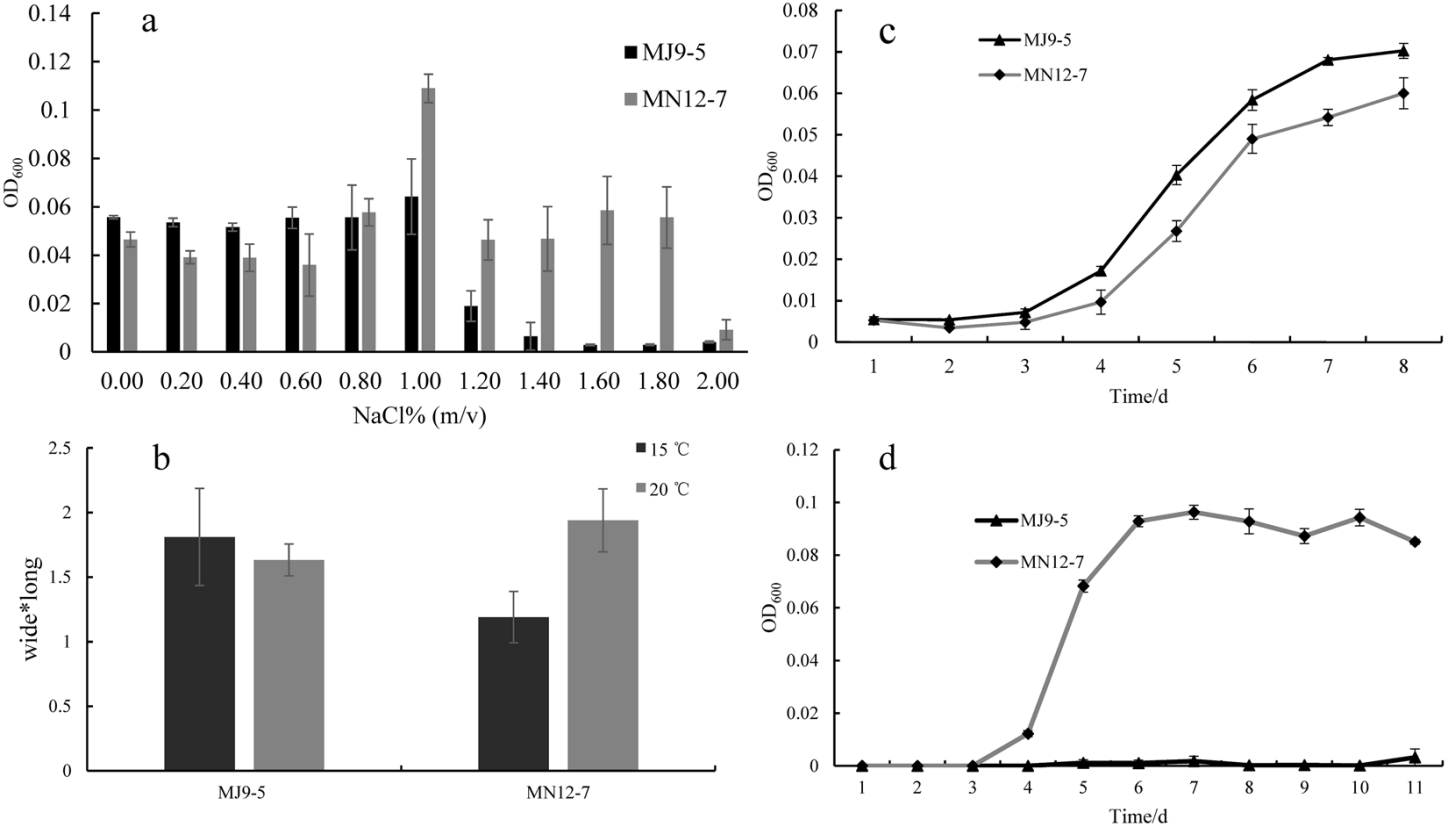
(**a**) NaCl tolerance of strains MJ9-5 and MN12-7 (**b**) cell sizes of strains MJ9-5 and MN12-7 after incubation at 15 °C and 20 °C; and growth of strains MJ9-5 and MN12-7 at (**c**) 4 °C and (**d**) 25 °C.

Strain MJ9-5 grew between −1 °C and 25 °C under laboratory conditions, with an optimum temperature range of 10 °C–15 °C on R2A media. Strain MN12-7 grew between 4 °C and 25 °C with an optimum temperature of 18 °C. The colonies were pink, semi translucent and convex for both strains MJ9-5 and MN12-7. Strain MJ9-5 tolerated 0–1.2% NaCl (w/v), with an optimum of 1%. Strain MN12-7 tolerated 0–1.8% NaCl (w/v), with an optimum of 1% (Fig. 1a).

After 4 days of incubation at 15 °C, the cell sizes of strain MJ9-5 were 1.00 ± 0.05 mm wide and 1.80 ± 0.27 mm long; when the incubation temperature was 20 °C, the cell sizes were 0.91 ± 0.09 mm wide and 1.80 ± 0.20 mm long. The cell sizes of strain MN12-7 at 15 °C were 0.71 ± 0.04 mm wide and 1.68 ± 0.22 mm long. In contrast with strain MJ9-5, when the incubation temperature was set at 20 °C, the cell sizes of strain MN12-7 increased to 0.86 ± 0.06 mm wide and 2.26 ± 0.29 mm long (Figs S1 and 1b).

Comparison of the growth curves for MJ9-5 and MN12-7 at 4 °C and 25 °C indicated that strain MJ9-5 is better adapted to low temperatures, but has a poor response to higher temperatures, with respect to strain MN12-7 (Fig. 1c,d).

Strains MJ9-5 and MN12-7 had similar utilization of the substrates in API 20 NE strips, but strain MJ9-5 was able to utilize glucose, mannose and maltose at 15 °C, while MN12-7 could not. Both strain MJ9-5 and MN12-7 were not able to utilize starch and glycogen (Table S1).

### Overview of genome

The genome sequence of strain MJ9-5 was 4,687,084 bp, with 97 contigs. It had 4,086 predicted genes, including three 5S rRNA, one 23S rRNA, one 16S rRNA, thirty-nine tRNA genes and four CRISPRs. The DNA G + C content of strain MJ9-5 was 38.53 mol%. The genome completeness of MJ9-5 and MN12-7 were nearly 100% (Table 1).

**Table 1:** Genomic and phenotypic characteristics of strains in the family Sphingobacteriaceae with a sequenced genome.

	Aeur	Asva	Mpal	Osit	Parc	Pbor	Pcry	Pgin	Pglu	Phep	Pkyu	Pory	Ppan	Psal	Scan	Spau	Sspi	Stha
Source	ice	soil	peat bog	wastes	soil	soil	cryoconite	soil	soil	soil	soil	soil	soil	soil	human	silkworms	human	blood
Location	TP	Arctic	Siberia	Crete	Arctic	Montana	Austria	Korea	TP	ND	Korea	Korea	Korea	ND	ND	India	ND	ND
Size (Mbp)	4.68	4.68	8.40	5.05	4.22	5.55	5.95	6.51	4.01	5.17	6.35	3.44	6.34	4.63	5.20	5.12	5.09	5.90
Completeness (%)	100	99.52	97.46	100	97.61	97.61	100	97.61	100	100	98.09	100	100	100	100	99.37	99.84	99.84
*CDSs	4086	4257	7729	4639	3766	4932	5171	5594	3481	4361	5373	3186	5396	4012	7792	4419	4425	5332
Specific genes	795	1138	3625	1958	1209	1101	1649	1105	999	986	1059	999	1441	1129	2144	1723	1399	1955
RNAs	42	43	67	55	42	55	86	57	46	66	57	43	52	65	72	81	55	72
RGT (°C)	−1–25	4–25	2–33	5–45	4–25	4–37	1–25	4–30	15–37	<37	4–40	15–35	4–30	ND	10–40	ND	<42	>42
OGT (°C)	10–15	18	20	28–32	18	30	20	ND	30	24	ND	30	25	25	18–30	ND	30	30
HWP (%)	2.6	3.1	6.5	3.2	3.0	2.9	4.1	3.2	2.6	4.1	3.7	1.3	6.2	3.7	2.6	5.2	3.2	3.9
NaCl (%)	0–1.2	0–1.8	<1	<3	0–2	ND	ND	0–2	0–3	ND	1–2	<2	0–1	ND	ND	ND	ND	ND
G + C (%)	38.5	38.2	42.9	44.6	36.9	38.4	38.8	38.7	34.4	42.1	40.5	37.8	38.4	36.6	37.3	40.9	39.8	43.6
CRISPRs	3	0	2	0	0	0	1	0	0	0	0	0	0	0	0	0	1	0
Cold shock	2	2	4	4	3	7	3	7	3	4	6	3	4	3	2	4	2	3
Heat shock	13	13	15	14	14	13	13	13	14	14	14	14	15	14	14	14	13	13

*Abbreviations: CDSs, coding sequences; RGT, range of growth temperature; OGT, optimal growth temperature; HWP, homology within proteomes; TP, Tibetan Plateau; ND, no data. Abbreviations for Sphingobacteriaceae strains are defined in Fig. 2 caption.

**Figure 2:**
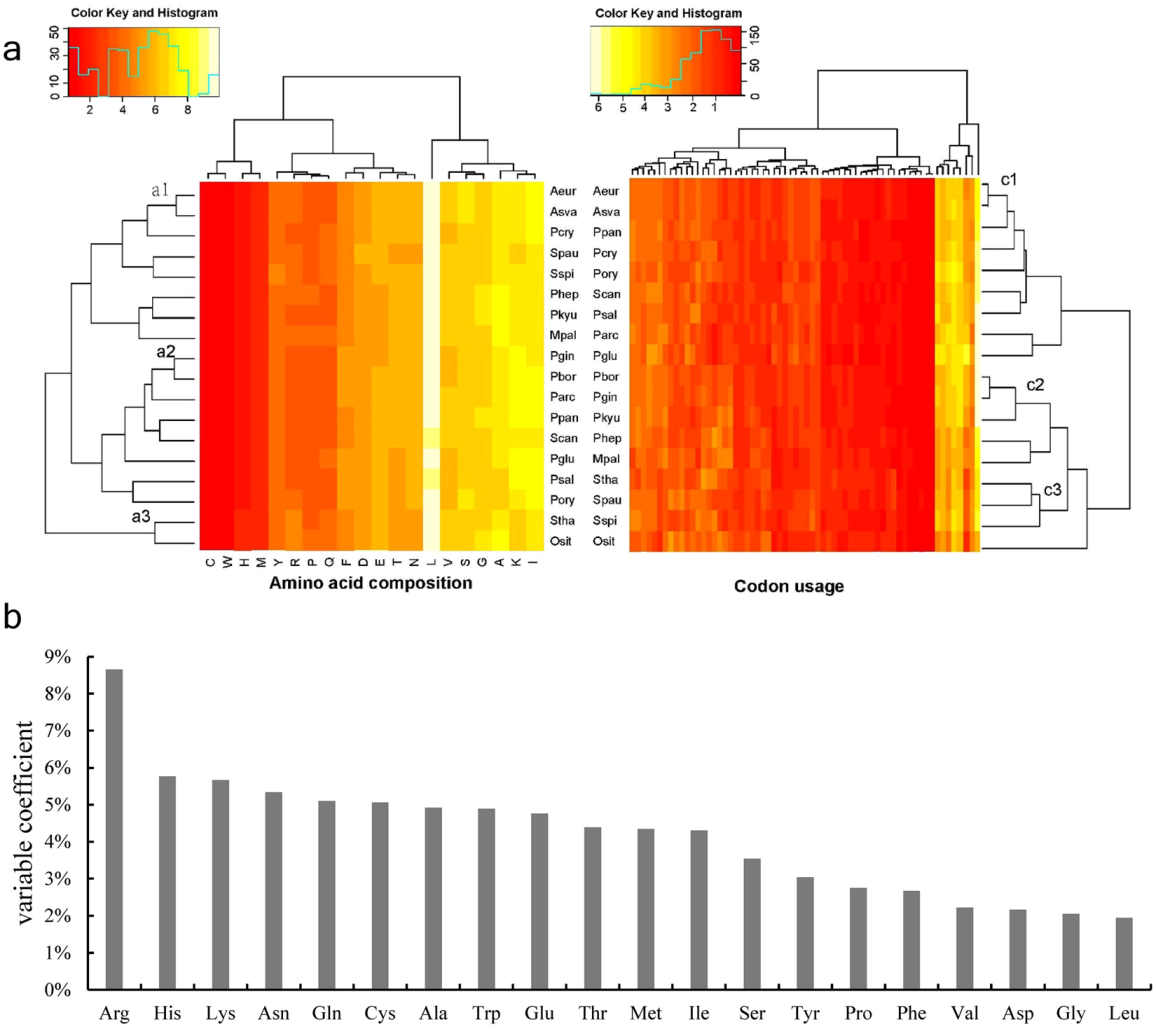
(**a**) Amino acid and codon usage heat maps. Key: Aeur, *Arcticibacter eurypsychrophilus* MJ9-5; Asva, *Arcticibacter svalbardensis* MN12-7; Mpal, *Mucilaginibacter paludis* DSM18603; Osit; *Olivibacter sitiensis* DSM17696; Parc, *Pedobacter arcticus* A12; Pbor, *Pedobacter borealis* DSM 19626; Pcry, *Pedobacter cryoconitis* PAMC 27485; Pgin, *Pedobacter ginsenosidimutans* KACC 14530; Pglu, *Pedobacter glucosidilyticus* DSM 23534; Phep, *Pedobacter heparinus* DSM 2366; Pkyu, *Pedobacter kyungheensis* KACC 16221; Pory, *Pedobacter oryzae* N7; Ppan, *Pedobacter panaciterrae* O48; Psal, *Pedobacter saltans* DSM 12145; Scan, *Solitalea canadensis* DSM 3403; Spau, *Sphingobacterium paucimobilis* HER 1398; Sspi, *Sphingobacterium spiritivorum* ATCC 33861 and Stha, *Sphingobacterium thalpophilum* DSM 11723). (**b**) Variable coefficients of amino acid composition between the 18 strains.

**Figure 3:**
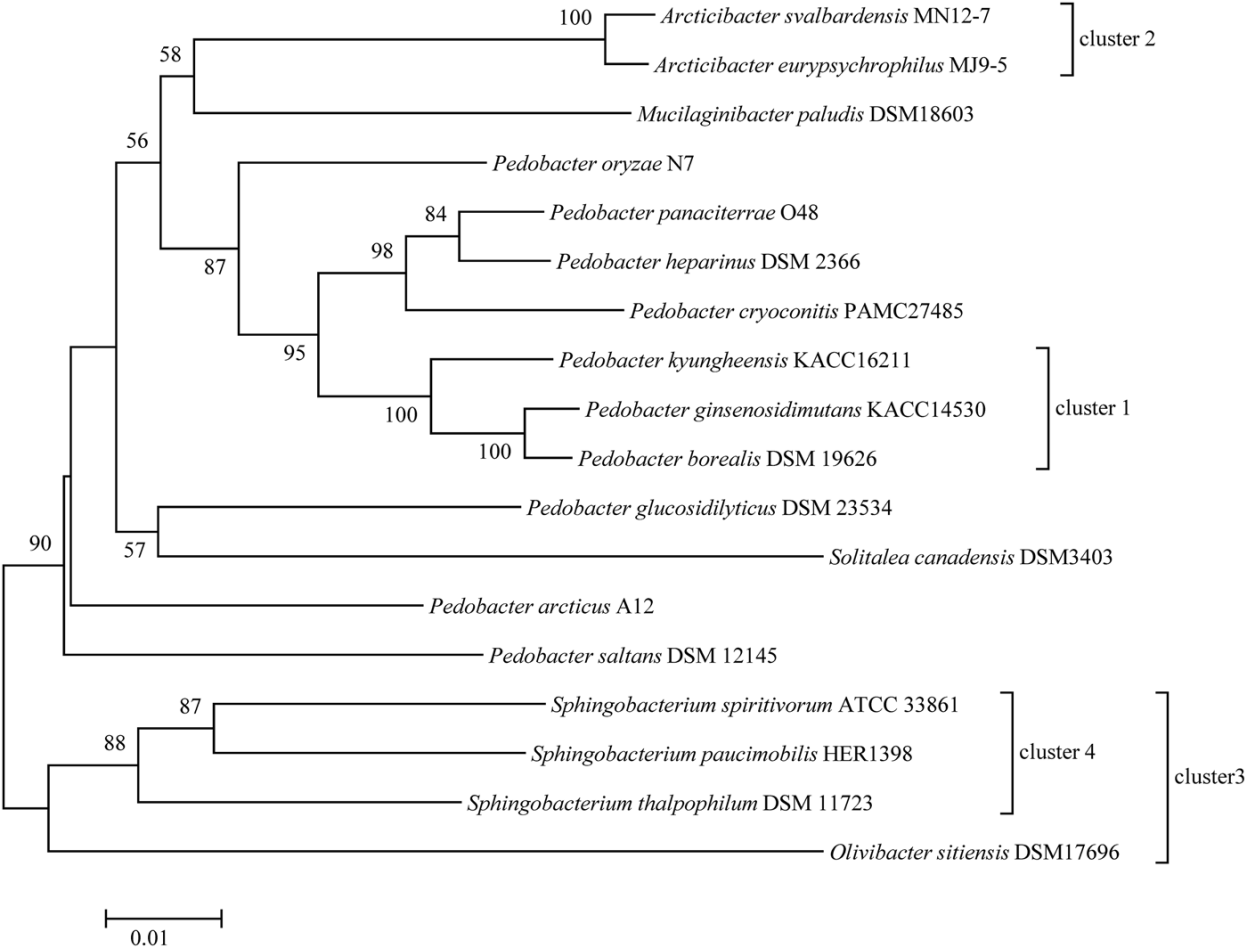
Phylogenetic relationships of the 18 strains analyzed, all 16S rRNA genes were extracted from each genome. Bar, 0.01 accumulated changes per nucleotide.

The codon usage and amino acid compositions of strains MJ9-5, MN12-7 and the 16 other reference strains from the family *Sphingobacteriaceae* with genomes available in GenBank, were analyzed. All 18 strains had similar codon usage and amino acid compositions, except for the amino acid arginine, the levels of which were apparently abundant in strains *Sphingobacterium thalpophilum* DSM 11723 and *Olivibacter sitiensis* DSM 17696, which had relatively higher upper limits of growth temperature (Fig. 2). Clade a1, c1, c2 and c3 in Fig. 2 were well reproduced in the phylogenetic tree created using the 16S rRNA gene (Fig. 3).

Confirmed CRISPRs were identified in four of the Sphingobacteriaceae genomes analyzed: strain MJ9-5, *Mucilaginibacter paludis* DSM18603, *Pedobacter cryoconitis* PAMC 27485, *Pedobacter glucosidilyticus* DSM 23534 and *Sphingobacterium spiritivorum* ATCC 33861. The number of confirmed CRISPR locations were: one in each of *P. cryoconitis* PAMC 27485 and *S. spiritivorum* ATCC 33861; three in strain MJ9-5; and nine in *M. paludis* DSM18603. The number of spacers ranged from 3 to 242. The lengths of direct repeat (DR) sequences ranged from 30 to 46 bps; no identical DR sequences were shared by the four species (Table 2).

**Table 2:** Information of confirmed CRISPRs from Sphingobacteriaceae strains.

	Number of CRISPRs	Length	DR Length^*^	Number of spacers	Spacer length
Aeur	CRISPR 1	3850	30	17	21–66
	CRISPR 2	229	30	3	36–37
	CRISPR 3	1195	30	57	35–71
Mpal	CRISPR 1	2697	46	35	29–30
	CRISPR 2	15894	30	242	34–39
Pcry	CRISPR 1	4900	46	64	29–30
Sspi	CRISPR 1	2697	46	34	30

*DR, direct repeats. Abbreviations for Sphingobacteriaceae strains are defined in Fig. 2 caption.

### Features in subsystems

Note that the genomes analyzed in the present were not complete except *P. heparinus* DSM2366; thus, observations maybe a product of incomplete assembly (genome completeness of each strain is presented in Table 1).

#### Genes associated with stress response

**Figure 4:**
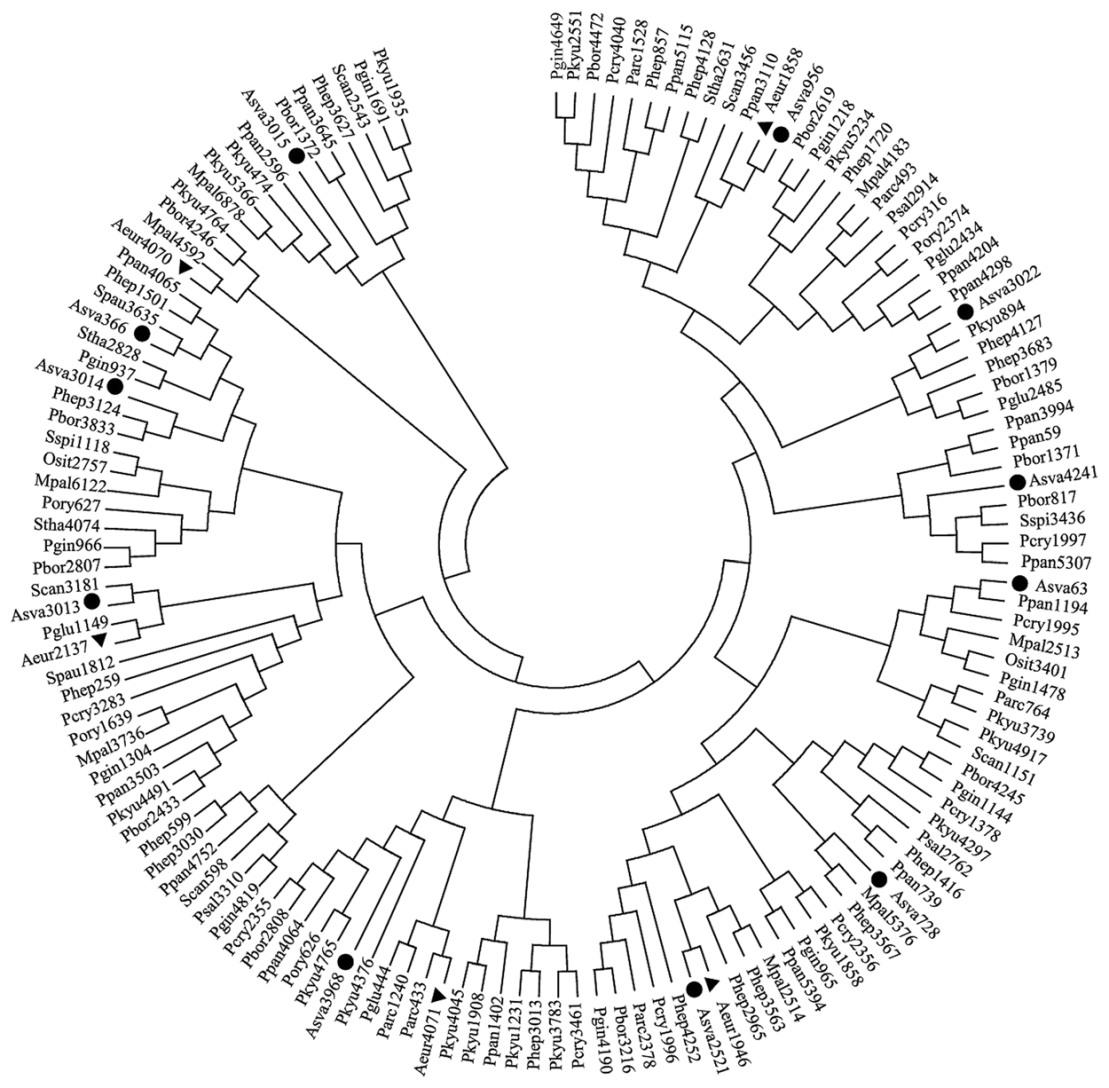
Phylogenetic relationships of genes related to osmotic regulation.

Genes affiliated with the ‘stress response’ category ranged from 53 in strain *O. sitiensis* DSM17696 to 93 in strain *P. kyungheensis* KACC 16221. Genes assigned to stress response subcategories, such as ‘cold shock’, ‘heat shock’, ‘detoxification’, ‘osmotic stress’, ‘periplasmic stress’ and ‘oxidative stress’ were identified in all of the 18 strains analyzed. Most of the cold and heat shock DnaK/DnaJ/GrpE chaperone system genes in the 18 strains formed similar clades as those constructed by the 16S rRNA genes (Figs 3, S2 and S3). Strains MJ9-5 and MN12-7 each had two cold shock genes and shared highly identical nucleotide sequences (Fig. S2). In the category ‘stress response’, 39 genes with different functions were absent from the genus *Arcticibacter*; these included an absence of genes coding for carbon starvation protein A, glycerol uptake facilitator protein, lactoylglutathione lyase, organic hydroperoxide resistance protein and outer membrane stress sensor protease DegQ. There were only five subcategories of genes unique to the genus *Arcticibacter*, which encoded rubrerythrin (two copies in strain MJ9-5), HflK and HflC proteins, glutathione peroxidase and D-tyrosyl-tRNA (Tyr) deacylase (equally shared by MJ9-5 and MN12-7). Osmotic related genes, in the ‘osmotic stress’ subcategory, were rare in strain MJ9-5 (Fig. 4).

In addition to glycerol, glutamate, betaine and choline, genes coding for trehalose and amino acids synthesis proteins are critical for salinity tolerance and osmotic adaption^3, 31^. The genomes of strains MJ9-5 and MN12-7 harbored 9 and 12 trehalose biosynthesis genes, respectively. Both of the two strains harbored six proline synthesis and five glutamine synthetase genes. This indicates that, the different salinity adaption profile of strains MJ9-5 and MN12-7 was more likely result from the asymmetric betaine, choline and trehalose metabolism.

#### Genes associated with monosaccharide metabolism

**Figure 5:**
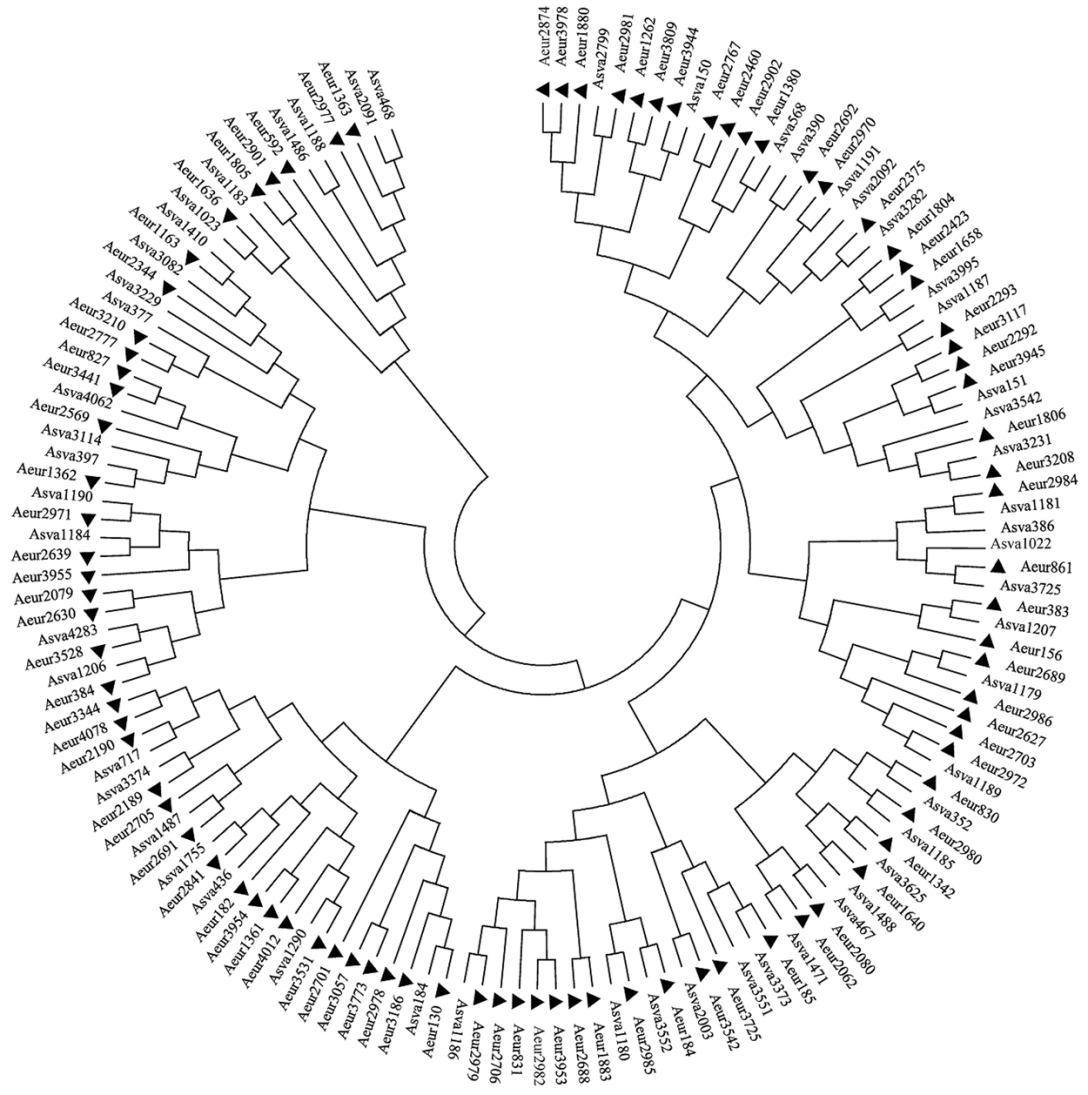
Phylogenetic relationships of genes related to monosaccharide metabolism.

Strain MJ9-5 was very divergent with 89 monosaccharide metabolism genes, while strain MN12-7 had 55 (Fig. 5). Phylogenetic analysis of the monosaccharide metabolism genes showed that branches with strain MN12-7 genes located in them also included genes from strain MJ9-5. However, some branches with genes from strain MJ9-5 in them had no genes from strain MN12-7, such as the branch composed of genes Aeur 1883, 2688, 831, 3777 and 3050. Monosaccharide-related genes were disproportionately increased with multiple copies, with respect to the relatively smaller genome size and fewer coding sequences in strain MJ9-5.

#### Presence and absence of genes in functional categories

Although not all of the genomes were completely sequenced, the absence of whole gene clusters may not entirely result from these incomplete genomes. All of the functional categories analyzed were represented in genes from each of the 18 strains analyzed, except foe category A (phages/prophages/transposable elements/plasmids) and Z (motility and chemotaxis). The strain with largest genome, *M. paludis* DSM18603, was very divergent with 135 genes assigned to category A, which was three times greater than the other species analyzed, indicating a critical role of the genome fraction in the genomic evolution of *M. paludis* DSM18603. For the *Arcticibacter* species, strain MJ9-5 had no genes assignable to category A, while strain MN12-7 had 11 genes. These results indicate that genes related with ‘phages/prophages/transposable elements/plasmids’ varied considerably between the Sphingobacteriaceae strains and even between *Arcticibacter* species. For strain MN12-7, all of the 11 genes devoted to category A belonged to the subcategory ‘transposable elements’ and originated through HGT. This indicates the bias of genes associated with transposable elements during the accumulation of DNA. Alternatively, the acquisition of transposable elements may be inhibited by the CRISPR-Cas system in strain MJ9-5, although it was recently argued that there is no evidence of the inhibition of HGT by the CRISPR–Cas system on evolutionary timescales^32^.

Genes that encode transporter proteins are crucial for growth and survival in extreme environments; they may be essential for importing metals and compatible solutes (needed for enzyme activity), nutrients (for combating starvation) and osmoprotectants^33–35^. The genome of strain MJ9-5 harboreds 48 related genes related to transporter proteins (Table S3), including nine cation transporter genes (Mg/Co/Ni/Cu), which are essential for enzyme activity; for example, magnesium transporter genes Aeur216, Aeur2371 and Aeur761, which are critical for the activity of DNA polymerase^36^.

### ANI of genomes and genome fractions assigned to category

**Figure 6:**
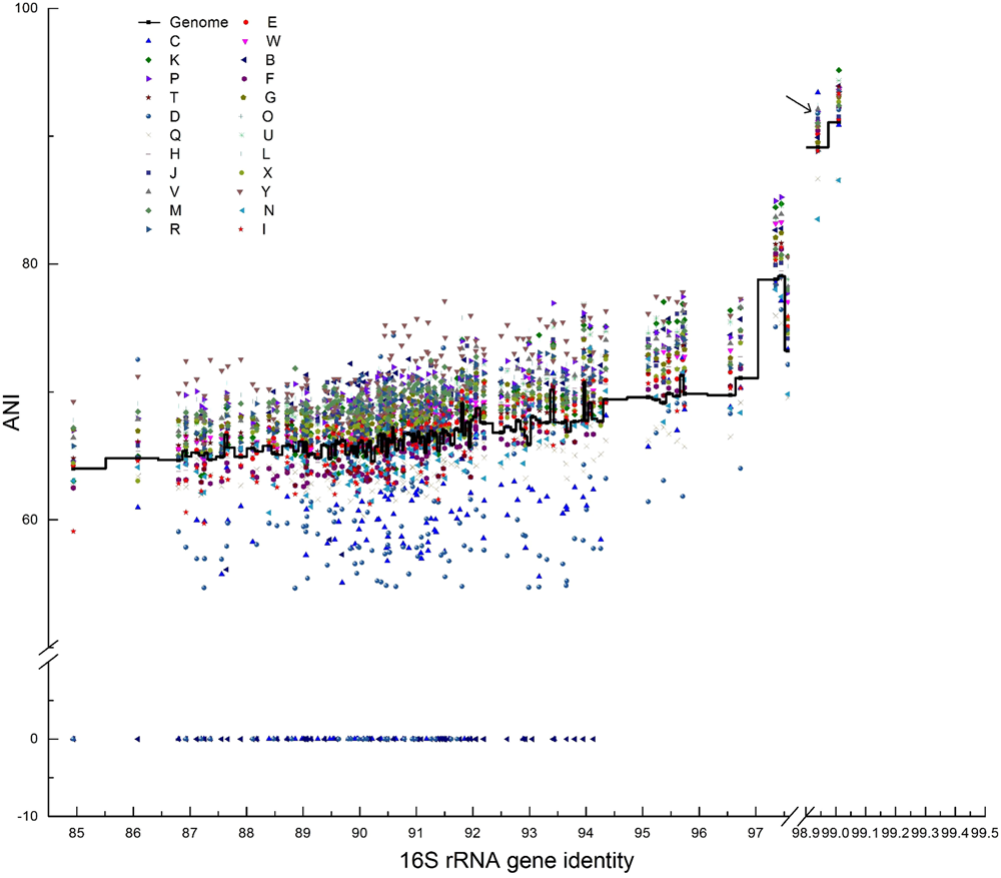
Relationships between 16S rRNA gene identity for all pairs of the reference genomes and the ANI of either the whole genome or the gene cluster (based on functional category); the arrow points to pairwise ANIs between strain MJ9-5 and MN12-7; pairwise comparisons with no hits were assigned as 0. Functional categories are: B, metabolism of aromatic compounds; C, iron acquisition and metabolism; D, dormancy and sporulation; E, carbohydrates; F, cell wall and capsule; G, DNA metabolism; H, potassium metabolism; I, phosphorus metabolism; J, regulation and cell signaling; K, nitrogen metabolism; L, protein metabolism; M, stress response; N, sulfur metabolism; O, fatty acids/lipids/isoprenoids; P, cell division and cell cycle; Q, membrane transport; R, virulence/disease/defense; S, miscellaneous; T, cofactors/vitamins/prosthetic groups/pigments; U, nucleosides and nucleotides; V, RNA metabolism; W, amino acids and derivatives; X, respiration and Y, secondary metabolism. The environmental impacts in two Arcticibacter strains including sixteen Sphingobacteriaceae species.

Pairwise ANI of the 18 genomes ranged from 64.00% to 91.08%, as shown as the step H center line in Fig. 6. Relatively higher pairwise ANI and 16S rRNA gene identity levels were observed for strains *P. ginsenosidimutans* KACC 14530 and *P. borealis* DSM 19626 (91.08%, 99.01%); MJ9-5 and MN12-7 (89.11%, 98.94%); *P. borealis* DSM 19626 and *P. kyungheensis* KACC 16221 (79.05%, 97.45%); *P. ginsenosidimutans* KACC 14530 and *P. kyungheensis* KACC 16221 (78.79%, 97.35%); and *P. heparinus* DSM 2366 and *P. panaciterrae* O48 (73.23%, 97.57%).

The genomes were split into 26 categories based on the RAST subsystem and the pairwise ANIs of 23 functional categories were analyzed (categories A, S and Z were not considered). Pairwise ANIs of the 23 functional categories ranged from 54.7% (‘dormancy and sporulation’ between *S. spiritivorum* ATCC 33861 and *Solitalea. canadensis* DSM 3403) to 95.2% (‘nitrogen metabolism’ between *P. ginsenosidimutans* KACC 14530 and *P. borealis* DSM 19626). Figure 6 shows that the ANI values of the functional categories were distributed in three regions. The upper region, above the genomic ANI line, contained genes from all 18 strains that were ascribed into the categories Y (secondary metabolism), P (cell division and cell cycle), G (DNA metabolism), U (nucleosides and nucleotides), L (protein metabolism), V (RNA metabolism) and M (stress response). The genes in these categories were relatively highly conserved. The ANI values of genes in categories Q (membrane transport) and N (sulfur metabolism) were all below the genomic ANI line and formed the middle cluster. It is interesting that where 16S rRNA identity was lower than 94.5%, genes in categories B (metabolism of aromatic compounds), C (iron acquisition and metabolism) and D (dormancy and sporulation) varied dramatically; no hits were detected between some strains, even within the same genus (the bottom cluster in Fig. 6).

### Horizontally transferred genes

A total of 744 and 1023 genes were identified as HGT genes in strains MJ9-5 and MN12-7, respectively (Fig. S4). These horizontally transferred genes belonged to 64 orders for strain MJ9-5 and 48 orders for strain MN12-7. In the annotation of the HGT genes, 744 genes from strain MJ9-5 were affiliated to 17 functional subsystems, while 1023 were affiliated to 19 functional subsystems for strain MN12-7. The categories ‘carbohydrates’, ‘DNA metabolism’, ‘nitrogen metabolism’ and ‘sulfur metabolism’ had most of the HGT genes from strain MJ9-5, while ‘DNA metabolism’, ‘regulation and cell signaling’, ‘membrane transport’ and ‘carbohydrates and potassium metabolism’ were the main categories containing HGT genes for strain MN12-7 (Fig. S5).

## Discussion

### Growth temperature and genome composition

It is important for psychrotolerant species to produce enzymes that perform effectively at low temperatures. This is especially relevant for strain MJ9-5, which was isolated from Muji glacier, a habitant which the annual average air temperature was lower than −10 °C. Previous studies suggested that psychrophilic bacteria and their mesophilic counterparts have different patterns of amino acid compositions, which usually vary in the composition of asparagine, serine and arginine^2, 10, 37^. Amino acid composition and codon usage analyses revealed that strains in the same family shared similar usage of most of amino acids and codons. Branches formed by descendants of MJ9-5 and MN12-7 were well represented in dendrograms of the 16S rRNA genes, amino acid composition and codon usage. Although strain MJ9-5 appears more adapted to cold temperatures and was less able to sustain growth up to 25 °C than MN12-7, there were no remarkable differences in genome composition. However, strains *O. sitiensis* DSM17696 and *S. thalpophilum* DSM 11723, which could grow at the highest temperatures (>42 °C) had apparently higher levels of arginine; they formed a unique clade in the dendrogram of amino acid composition and had the highest variable coefficient of arginine composition among the 19 amino acids analyzed (Fig. 2a). Strains *P. borealis* DSM 1962, *Pedobacter oryzae* N7 and *S. spiritivorum* ATCC 33861 had similar optimal growth temperatures to those of strains *O. sitiensis* DSM17696 and *S. thalpophilum* DSM 11723 (of 30 °C), but no distinct bias of arginine was observed. Furthermore, while strains MJ9-5 and MN12-7, and *P. panaciterrae* O4 and *S. canadensis* DSM3403, had different minimal growth temperatures, they had similar amino acid compositions, revealing that the bias of amino acids may be less related to the lowest growth temperature. It is worth noting that strains *O. sitiensis* DSM17696 and *S. thalpophilum* DSM 11723 (with the highest maximum growth temperature) also had divergent G + C contents of 44.6% and 43.6%, respectively, which were higher than the other 16 strains (all lower than 43%). A variation of asparagine and serine was not detected in the present study, revealing that adaption to cold environment via the amino acid composition shifting may not occur in all biased amino acid residues for a certain bacteria taxon. In addition, a heat shock related gene for a methylthiotransferase, which may be critical for bacteria to thrive in temperate environments, like GrpE, was absent from strains MJ9-5 and MN12-7. These differences may be the reason why the *Arcticibacter* species are restricted to the cold Polar Regions (the Arctic and ‘the third pole’, the Tibet Plateau).

### Growth temperature and cold and heat shock genes

The physiological differences reflected by the opposite responses to temperature and the lack of phylogenetic analysis of genes related to cold and heat shock genes provided scope for further analysis of the stress response related genes in strains MJ9-5 and MN12-7, along with the reference strains. Cold shock proteins are induced upon cold shock and are thought to bind a single stranded RNA motif, resulting in reduced secondary structure formation in the RNA and thus increased translation efficiency^38^. It is important for bacteria to be able to synthesize proteins at low temperatures when present in environments with extreme low temperatures^38, 39^. The cold shock genes clustered as Aeur/Asva and Pbor/Pgin/Pkyu (Fig. S2) and these strains constituted a single cluster on the 16S rRNA tree; the related strains were able to grow at 4 °C. However, the clusters for Osit and Stha, related strains that grew at high temperatures, were not well represented in Figs 2 and 3. For instance, the cold shock genes Osit3179, Osit3507 and Stha3076 and Stha4135 were located on different branches (Fig. S2). Strains MJ9-5 and MN12-7 were both able to tolerate relatively low temperatures and there were no remarkable differences in their ‘cold shock’ genes. Actually, genes Aeur1235 and Asva3755 and Aeur1234 and Asva3756 encode the same amino acid sequence of cold shock gene (Fig. S2). The results revealed that cold shock associated genes are conserved in the cold adapted bacteria, while they are diverse in strains that tolerate high temperatures. For the two cold tolerant strains, the conserved cold shock genes also indicated different adaptabilities to the minimal temperatures resulting from other regulatory factors.

Heat shock system machinery exerts two different chaperone functions: refolding the denatured proteins and prevention of the aggregation of proteins^40^. For instance, in the DnaK/DnaJ/GrpE molecular chaperone system, the heat shock response is originally stimulated by differential temperature dependencies in the activity of DnaJ, which result in the hydrolysis of DnaK-bound ATP and the activity of GrpE^40^. In contrast, for cold shock genes, there were differences in the DnaK/DnaJ/GrpE chaperone system between the strains MJ9-5 and MN12-7, which had different growth patterns at their maximum growth temperatures. The genes of strains MJ9-5 and MN12-7 encoded chaperone protein DnaJ and Dnak and were located in the same branches. However, strains *O. sitiensis* DSM17696 and *S. thalpophilum* DSM 11723, which were more tolerant to higher temperatures, harbored only one copy of the GrpE gene. Within the genus *Arcticibacter*, strain MN12-7, which was more tolerant of high temperatures also harbored one GrpE gene, while strain MJ9-5 had two. These results indicate the efficiency of DnaK/DnaJ/GrpE chaperone system for the 18 strains may not be based on multiplicity of genes.

### Salinity tolerance and osmotic related genes

Study of a cold adapted Antarctica bacterium, *P. haloplanktis* TAC125, showed an enhanced ability to grow in the absence of NaCl at low temperatures, which indicated adaption to ice or melting ice water^35^. Strain MJ9-5 had a relatively narrow range of salinity tolerance in comparison with MN12-7, but it grew better than MN12-7 at low salinities. The analyses of osmosis related genes revealed a different pattern to the genome composition, cold and heat shock related genes. Most of the clades in 16S rRNA gene tree could not be observed in the dendrogram of ‘osmotic stress’ related genes, revealing a highly diversity of osmosis related genes. Except for two pairs of homologous genes, the *Arcticibacter* species MJ9-5 and MN12-7 shared no other homologs. The ‘choline and cetaine uptake’ and ‘betaine biosynthesis associated’ genes in strain MN12-7 were absent from strain MJ9-5. In addition, three compatible solute biosynthesis proteins in the ‘trehalose biosynthesis’ system, one alpha-amylase, one trehalose synthase and one glucoamylase, were not encoded in the MJ9-5 genome. Differences in salinity between each of the habitats may be more remarkable than those of temperature, for strains MJ9-5 and MN12-7. This may also be the case for most of the reference strains. In fact, Muji glacier’s concentration of Na^+^ was only one fifth of that in the Svalbard soil^19^. The relatively conserved growth temperature related genes and the higher diversity of osmosis related genes might indicate different levels of environmental pressure in driving the gene diversity^12^.

### Carbon source utilization and monosaccharide related genes

The death of cells that are adapted to hostile conditions in glacial ice probably occurs due to the exhaustion of available nutrients^41^ and strains that acquire the ability to utilize a larger number of carbon sources through genetic plasticity may have higher chances of survival. Studies revealed that bacterial isolates from cold environments, such as glacier cryoconite holes and the Arctic ocean, preferred simpler forms of carbohydrates^42, 43^. Strain MJ9-5 was able to utilize monosaccharides, such as glucose and mannose at 15 °C, while strain MN12-7 could not, which might indicate that strain MJ9-5 prefers simpler forms of carbohydrates. Since MJ9-5 was not able to utilize starch or glycogen, it is hypothesized that MJ9-5 could use monosaccharides may resulting from direct use of simple forms of carbohydrates.

The monosaccharide related genes in strain MN12-7 all had homologs in strain MJ9-5. However, strain MJ9-5 (isolated from an ice core) was very divergent, with 89 monosaccharide metabolism genes, while strain MN12-7 had 55 such genes. This was unusual, since most of the functional genes found occurred in pairs, with limited differences in their number (Figs 4, S2 and S3). Monosaccharide related genes were disproportionately increased, with multiple copies, relative to the relatively smaller genome size and fewer coding sequences of strain MJ9-5. This indicates that the apparent preference of monosaccharides by strain MJ9-5 might have been enabled by an enrichment of monosaccharide related genes, via multiplicity.

### Different diversion rates of genome fractions assigned to categories

In bacteria with a large number of genes, the evolution of those genes is heterogeneous^44^. An ANI value represents the relatedness between two genomes, it can be used to measure the genetic distance between genomes^2^. Here, the ANI values were further used to measure the genetic distance between gene clusters in each functional category. The relative evolutionary rates of genes in the different functional categories were assessed using the ANI values of the genomes as references. Figure 6 shows apparent conservation of genes assigned to category Y (secondary metabolism). Secondary metabolites, unlike primary metabolites, are not directly involved in the growth or reproduction of bacteria and the absence of secondary metabolites does not result in immediate damage. On the contrary, genes devoted to D (dormancy and sporulation), which are directly involved reproduction and the response to environmental stress, were very divergent.

Genes devoted to category B (metabolism of aromatic compounds) were very diverse between strains *P. borealis* DSM 19626 and *P. ginsenosidimutans* KACC 14530, and between *P. glucosidilyticus* DSM 23534 and *P. heparinus* DSM 2366, but were conserved between strains *P. panaciterrae* O48 and *P. heparinus* DSM 2366, and between *M. paludis* DSM1 8603 and*. P. oryzae* N7. The different distribution patterns of genetic distance measured by the ANI levels of the genome fractions assigned to ‘secondary metabolism’, ‘dormancy and sporulation’ and ‘metabolism of aromatic compounds’ verified the heterogeneous evolution of genes under environmental pressure.

### Origin and function assignment of HGT genes

Studies have argued that HGT has been critical for the evolution of bacterial genomes in their adaption to extreme environments. However, the origin and function of HGT genes have rarely been considered^3, 45, 46^. Bacteria can exchange genes with ambient environments via HGT, mediated by viruses, plasmids and other elements^4^. HGT has been proposed to be relatively high in low temperature environments^47^. Although the number of HGT genes in strain MJ9-5 was less than that in strain MN12-7, the HGT genes in MJ9-5 were more diverse in origin than those in strain MN12-7. This indicates a relatively complicated and high level of interaction between related and unrelated organisms in the glacier habitats. This interpretation is also supported by the fact that more HGT genes originated from outside of the phylum Bacteroidetes in strain MJ9-5 than in MN12-7.

The acquisition of novel metabolic capabilities via the expression of foreign genes contributes to the capacity of bacteria to adapt to new environments and bacteria can benefit from functional HGT^3, 48^. The percentages of HGT genes in the functional categories K (nitrogen metabolism), N (sulfur metabolism), B (aromatic compounds metabolism) and I (phosphorus metabolism) differed dramatically between strains MJ9-5 and MN12-7 (Fig. S5). This is consistent with the results that the ANI values of the gene clusters in the categories K, N and B were lower between strain MJ9-5 and MN12-7 than they were between other strains in the family Sphingobacteriaceae. The category with the highest percentage of HGT genes in strain MJ9-5 was K (nitrogen metabolism), while it was B (aromatic compounds metabolism) in strain MN12-7. This indicates that strain MJ9-5 preferentially acquired novel metabolic capabilities for nitrogen metabolism, while strain MN12-7 preferentially acquired the ability to metabolize aromatic compounds. It is worth noting that no genes in category I (phosphorus metabolism) were HGT genes in strain MJ9-5, while 30% of the genes were HGT genes for category I in strain MN12-7. The different patterns of acquisition of novel metabolic capabilities may have occurred due to the different environmental pressures imposed by the glacier on the inland Tibetan Plateau compared with the soils on the coastal Svalbard. With respect of the soils in Svalbard, organisms in the glacier on the northwest Tibetan Plateau were less likely to be exposed to phosphorus and aromatic compounds; because concentrations of total PAHs (polycyclic aromatic hydrocarbons) in the soil close to Muji glacier were ~12.6 ng/g, compared with over than 40 ng/g in the Svalbard soil^19, 49^.

From the discussion of the relationship between growth temperature and genome composition, cold shock and heat shock genes, it can be concluded that, genomic adaption characteristics were more obvious when grouping the strains by their upper limit growth temperature rather than by their minimal or optimal growth temperature. The improper grouping principle may result in a belief that there are few general adaption characteristics that are suitable for all cold adapted taxa.

## Conclusion

Methods on the analysis of the relative conservation of gene clusters, with respect to whole genomes, were introduced in the present study; these help us to understand the variation of genes based on their functional classification, during the differentiation of species. When the genes were split into their different functional categories, some interesting clusters formed. Categories Y, P, G, U, L, V, and M all had ANI values higher than the genomic ANI values, suggesting that genes associated with these categories were relatively highly conversed. In contrast, genes in categories Q and E were likely less conversed. Inverse patterns of the genes in categories C, D and N, were observed for the *Arcticibacter* species in comparison with the other 16 reference strains; there were no genes in the *Arcticibacter* species associated with motility and chemotaxis. The genome analysis showed that strains MJ9-5 had genetic advantages to adapt to ice core environment. The two main different environmental pressures, salinity and nutritional level, may play an important role in shaping the genomic profile of strains MJ9-5 and MN12-7.

## Electronic supplementary material

Supplementary materials
