# Supplementary material for: Comparative genomic analysis reveals the environmental impacts on two Arcticibacter strains including sixteen Sphingobacteriaceae species
Source: Sci Rep. 2017 May 17;7:2055. doi: 10.1038/s41598-017-02191-4 (PMC5435697; doi:10.1038/s41598-017-02191-4)
Supplement: Supplementary file 1 — Supplementary materials [file 41598_2017_2191_MOESM1_ESM.pdf]

Scientific Reports

Comparative genomic analysis reveals the environmental impacts on two Arcticibacter strains including sixteen Sphingobacteriaceae species

Liang Shen[1, 2, 3], Yongqin Liu[1, 2*], Baiqing Xu[1, 2], Ninglian Wang[2, 4], Huabiao Zhao[1, 2], Xiaobo Liu[1] and Fei Liu[5]

[1]Key Laboratory of Tibetan Environment Changes and Land Surface Processes, Institute of Tibetan Plateau Research, Chinese Academy of Sciences, Beijing, 100085, China

[2]CAS Center for Excellence in Tibetan Plateau Earth Sciences, Beijing, 100085, China

[3]University of Chinese Academy of Sciences, Colledge of Resources and Environment, Beijing, 100049, China

[4]College of Urban and Environmental Science, Northwest University, Xian, 710069, China

[5]Institute of Microbiology, Chinese Academy of Sciences, Beijing, 100101, China

*Author for correspondence:

Yongqin Liu, yqliu@itpcas.ac.cn, Institute of Tibetan Plateau Research, Chinese Academy of Sciences, Beijing, 100101, China

Legends to the Supplementary figures and tables

Figure S1. Transmission electron micrography of cells of strains MJ9-5 and MN12-7 at 15° C and 20 °C.

Figure S2. Phylogenetic relationships of genes related to cold shock.

Figure S3. DnaK/DnaJ/GrpE molecular chaperone system of the 18 strains (a) chaperone protein DnaJ; (b) chaperone protein Dnak; (c) heat shock protein GrpE.

Figure S4. Origin of HGT genes observed in strains MJ9-5 and MN12-7.

Figure S5. Percentage of HGT genes in each functional category, in strains MJ9-5 and MN12-7.

Table S1. Differential carbon source utilization properties of strains MJ9-5 and MN12-7 at 15°C.

Figure S1

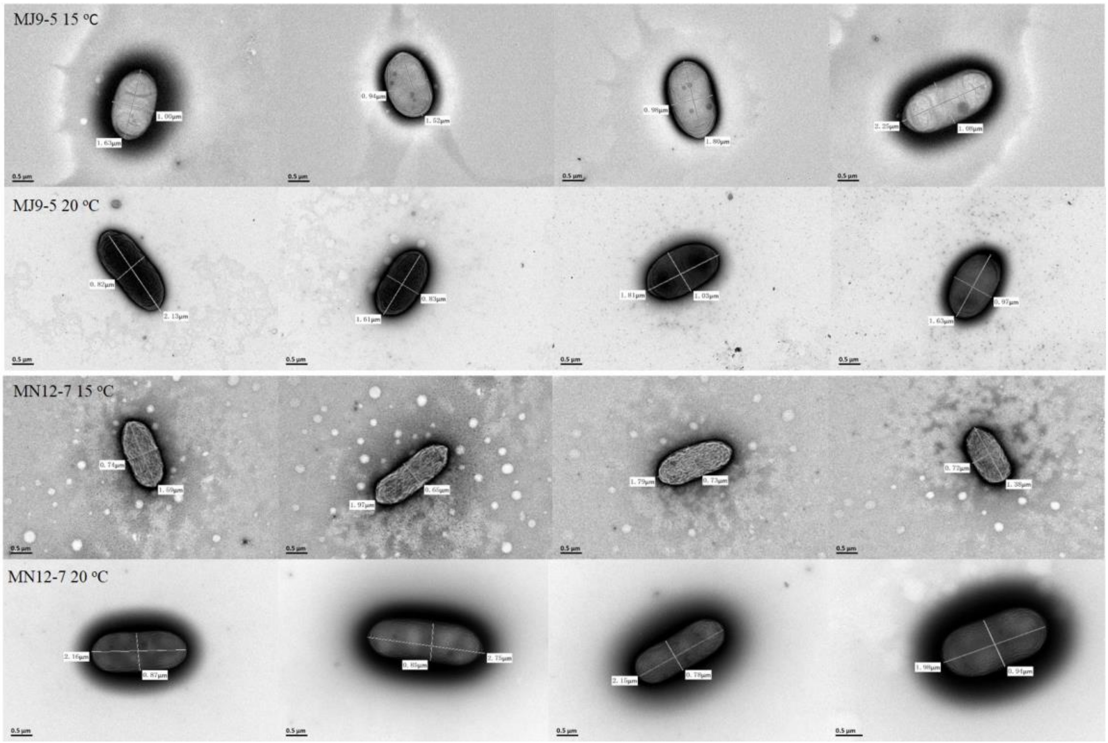

Figure S2

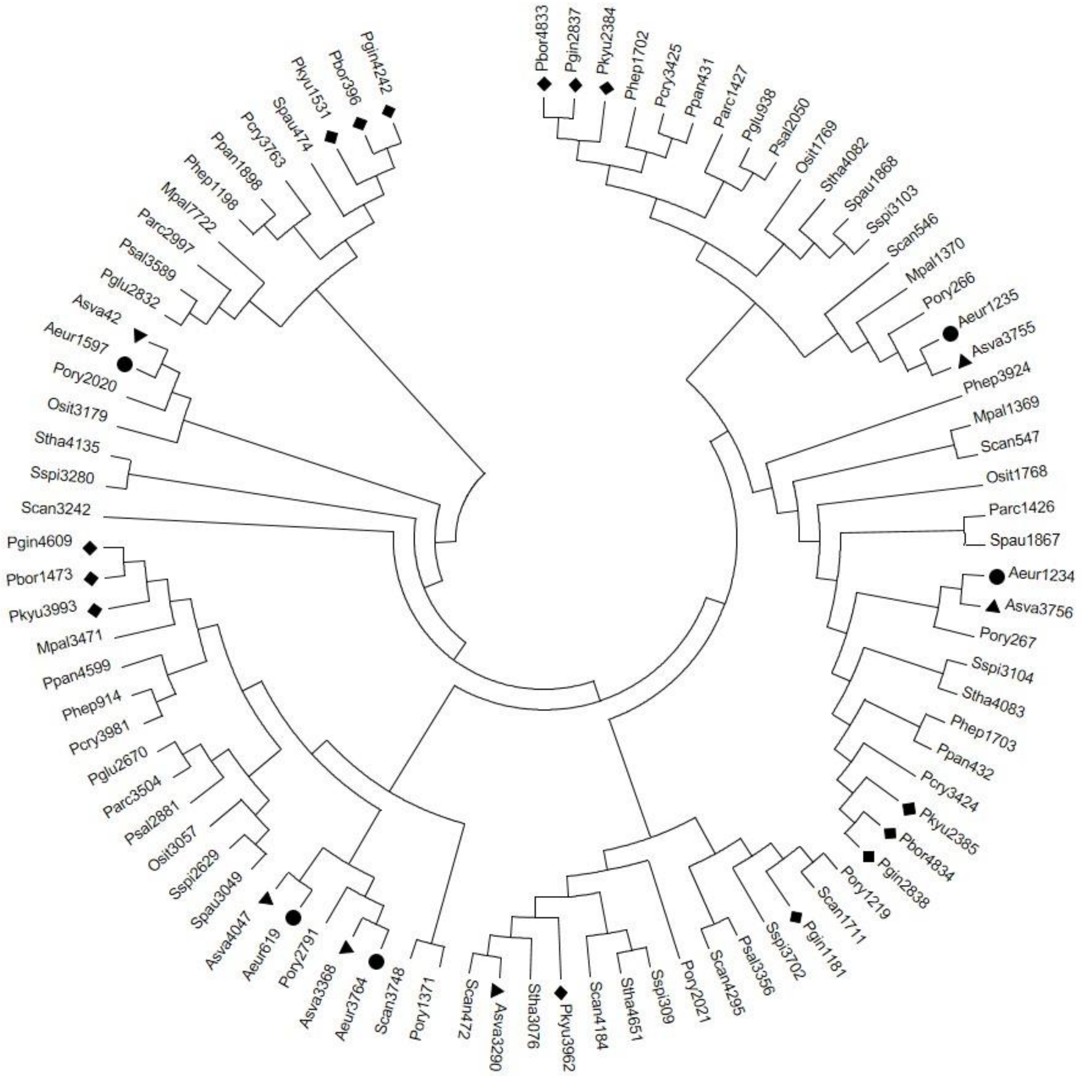

Figure S3

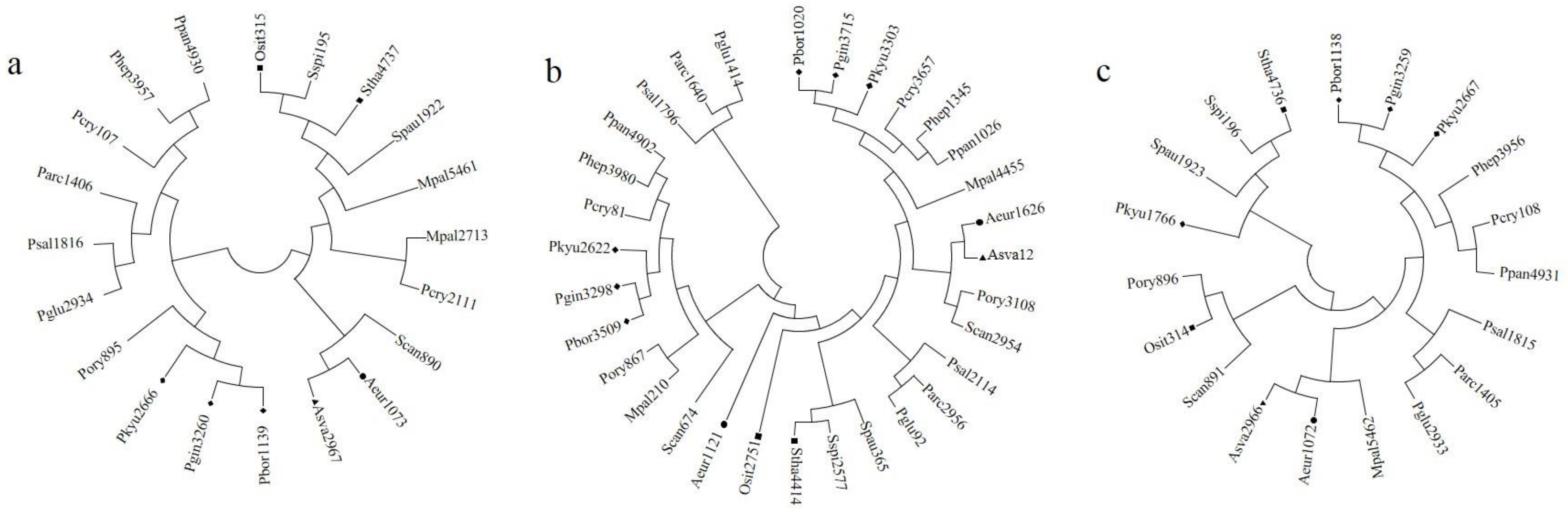

a
Ppan4930
Osit315
Sspi195
Stha4737
Phep3957
Pcry107
Spau1922
Mpal5461
Parc1406
Mpal2713
Psal1816
Pcry2111
Pglu2934
Scan890
Pory895
Aeur1073
Pkyu2666
Pgin3260
Pbor1139
Asva2967
b
Pbor1020
Pgin3715
Pkyu3303
Pglu1414
Parc1640
Psal1796
Pcry3657
Phep1345
Ppan1026
Ppan4902
Phep3980
Mpal4455
Pcry81
Aeur1626
Pkyu2622
Asva12
Pgin3298
Pory3108
Pbor3509
Scan2954
Pory867
Psal2114
Mpal210
Parc2956
Scan674
Pglu92
Aeur1121
Osit2751
Stha4414
Sspi2577
Spau365
c
Stha4736
Pbor1138
Pgin3259
Sspi196
Pkyu2667
Spau1923
Phep3956
Pkyu1766
Pcry108
Ppan4931
Pory896
Osit314
Psal1815
Scan891
Parc1405
Asva2966
Aeur1072
Mpal5462
Pglu2933

Figure S4

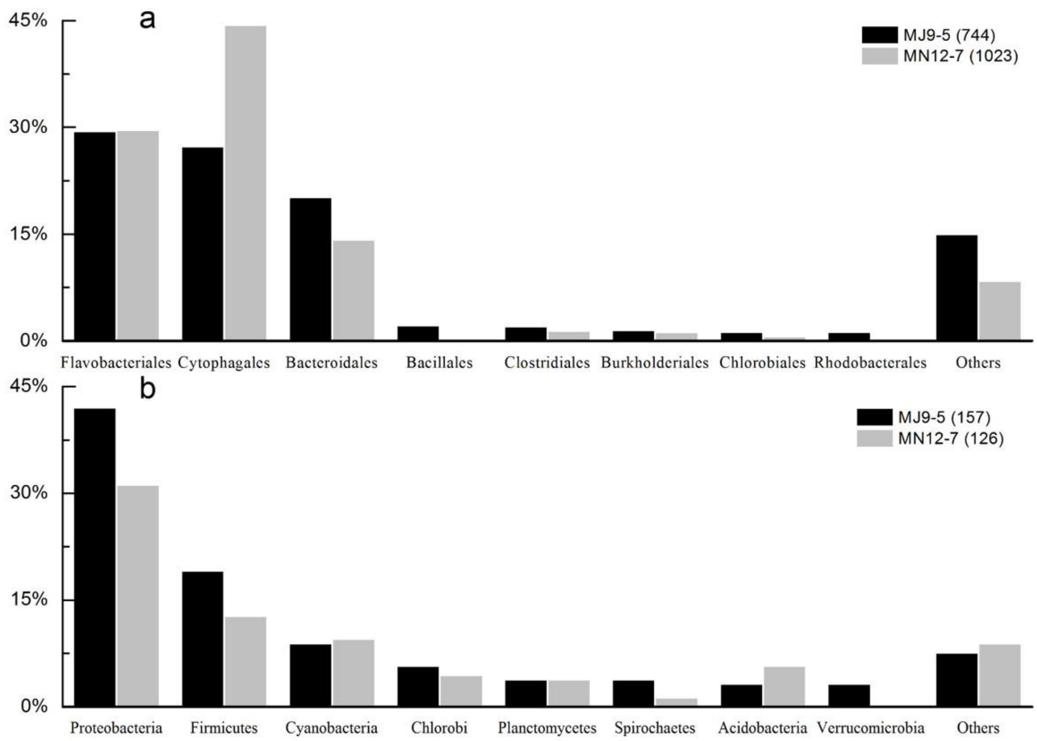
a
45%
30%
15%
0%
MJ9-5 (744)
MN12-7 (1023)
Flavobacteriales
Cytophagales
Bacteroidales
Bacillales
Clostridiales
Burkholderiales
Chlorobiales
Rhodobacterales
Others
b
45%
30%
15%
0%
MJ9-5 (157)
MN12-7 (126)
Proteobacteria
Firmicutes
Cyanobacteria
Chlorobi
Planctomycetes
Spirochaetes
Acidobacteria
Verrucomicrobia
Others

Figure S5

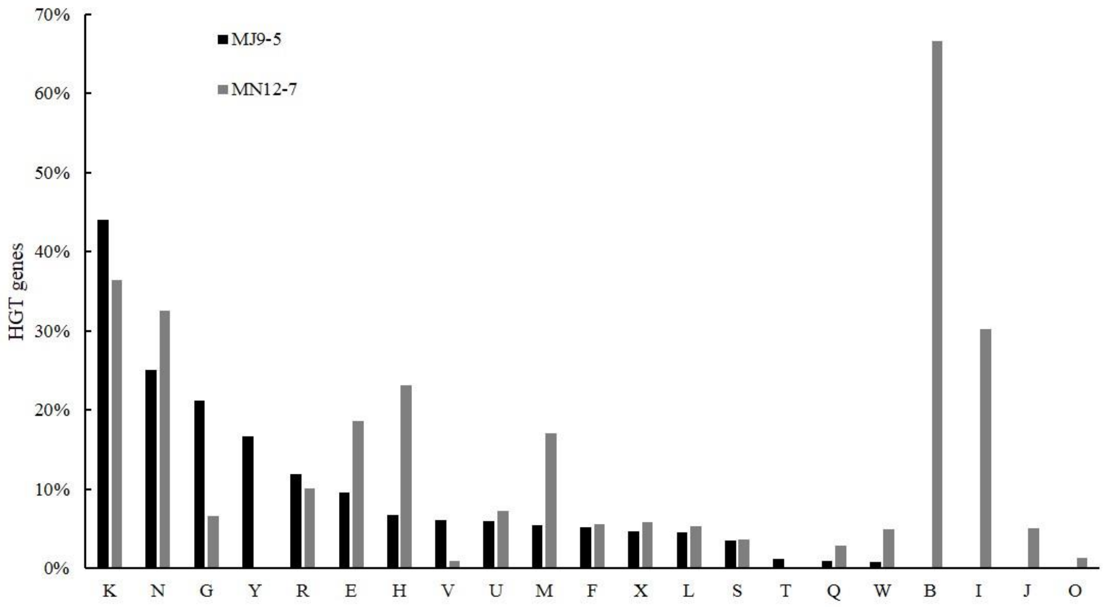

Table S1

	MJ9-5	MN12-7
Glucose	+	-
Arabinose	+	+
Mannose	+	-
Mannitol	-	+
N-b-glucosamine	+	+
Maltose	+	-
Gluconate	-	-
Capric acid	-	-
Adipic Acid	-	-
Malic acid	-	-
Citric acid	-	-
Phenylacetic acid	-	-
Starch	-	-
Glycogen	-	-

Supplementary material M1 Custom made perl scripts

```
#!/usr/bin/perl -w
use Getopt::Long;

=head1 Name
        animore.pl
=head1 Description

        This program calculates Average Nucleotide Identity (ANI) based on genomes of a pair of prokaryotes.
=head1 Usage

        animore.pl --ani ANI.pl --fd formatdb --bl blastall --od output directory --fsd fna files directory --help (optional)
        Arguments explained
        ani: ANI.pl path
        bl: Directory of blastall excecutable file
        fd: Directory of BLAST formatdb excecutable file
        od: output directory
        fsd: fna files directory
        help: print this help information
=head1 Example
        animore.pl -ani /home/ftpuser/vpdn/code/ANI.pl -bl /usr/bin/blastall -fd /usr/bin/formatdb -od /home/ani/result -fsd /home/ani/fnas
=cut
my ($ani,$fsd,$od,$fd,$bl,$hl);
GetOptions(
        "ani=s" => \$ani,
        "fsd=s" => \$fsd,
        "od=s" => \$od,
        "fd=s" => \$fd,
        "bl=s" => \$bl,
        "help" => \$hl
                );

die `pod2text $0` unless $ani && $fsd && $od && $bl && $fd;
die `pod2text $0` if $hl;

unless(-d $od){`mkdir $od`;}

$pathlast = (split //,$fsd)[-1];
if ($pathlast ne "\/") {
        $fsd = "${fsd}\/";
}
```

```
my @allfiles = `ls $fsd`;
my $filenum = @allfiles;
my $fileindex = 0;

for my $file (@allfiles) {
	$fileindex += 1;
	my $cmpindex = 0;
	chomp($file);
	for my $cmpfile (@allfiles) {
		chomp($cmpfile);
		$cmpindex += 1;
		print "${cmpfile} ${file} \n";
		if ($cmpfile eq $file) {
			next;
		}
		$mycmpresult = `perl $ani \-bl $bl \-fd $fd \-qr ${fsd}${cmpfile} \-od ${od} \-sb
${fsd}${file}`;
		$myresult = (split /\s+/,$mycmpresult)[3];
		chomp($myresult);
		#$myresult = $mycmpresult;
		$mydata[$fileindex][$cmpindex] = $myresult;
	}
}

for my $i (1..$filenum) {
	for my $j (1..$filenum) {
		if ($j < $i) {
			print "$mydata[$j][$i] ";
		}
	}
	print "\n";
}


#!/usr/bin/env perl

use strict;
use warnings;
use Getopt::Std;



my %opts=();
getopts('i:f:o', \%opts);
```

```
if(! defined ($opts{f}) || !defined ($opts{i})){
	warn "Usage: perl $0 -f in.fas -i id.txt\n";
	warn "    options:\n";
	warn "          -f    input fasta file\n";
	warn "          -i    only ouput sequence whose id found in this file\n";
	warn "          -o     keep sequence order as it in id file specified by -i \n";
	warn "\n";
	exit(0);
}

my %target=();
open my $ID,'<', $opts{i} or die "failed to open file \"$opts{i}\"\n";
my $num=0;
while(<$ID>){
	chomp;
	s/\r$//g;
	s/\n$//g;
	s/\s+$//g;
	next if (/^\s+$/);
	$target{$_}=++$num;
}
close $ID;

open my $FAS,'<',$opts{f} or die "failed to open file \"$opts{f}\"\n";
my $rand_str='';
if(defined $opts{o}){
	my @str=("A".."Z", "a".."z",0..9);
	$rand_str = join '', map $str[rand @str] , 1 .. 10;
}
$/='>';
while(<$FAS>){
	$_=~s/>$//;
	next if (/^\s+$/);
	if($_=~/^(\S+)/){
		my $id=$1;
		if(defined $target{$id} ){
			$_=~s/\s+$//;
			if (defined $opts{o}){
			open my $fh,'>', $rand_str.".".$target{$id}.".fas";
			print $fh ">$_\n";
			close $fh;
			}
			else{
			print ">$_\n";
```

```
			}
		}
	}
}

if(defined $opts{o}){
	foreach my $i (1..$num){
		open my $fh2,'<', $rand_str.".".$i.".fas";
		while(<$fh2>){
			print;
		}
		close $fh2;
		unlink $rand_str.".".$i.".fas";
	}
}

#!/usr/bin/env perl

# split fasta file to individual entry per file
# by zhuwei@big.ac.cn @2016-06-07
#
use strict;
use warnings;
use Getopt::Long;

my $INFILE;
my $mode=0;
my $IDFILE;
GetOptions ("fasta=s" => \$INFILE,
			"id=s"      => \$IDFILE,
			"mode=i"    => \$mode)
	or die("Error in command line arguments\n");

sub usage {
print STDERR <<EOF;
    usage: perl $0 --fasta your_fasta_file
	options:
		--fasta [FILE] a fasta file to process
		--id        [FILE] put your target sequence name in this file
		--mode    [INT]    process mode; possible value: 0, or other positive number
		                    if set to 0: each sequence would be ouptuted to individual file;
		                    if set to other number, sequence would be outputed to file
		                    based in their first INT    number of character in the sequence name
		                    eg. a sequence with name "ABCDEF" would be outputed to ABC.fas
```

```
if --mode set to 3

EOF
exit;
}
usage if (! defined $INFILE);
if ($mode <0){
	die "	--mode must be set to a number greater than 0\n";
}
open my $FAS,'<',$INFILE or die (" failed to open file: $INFILE\n");

my %target=();
if(defined $IDFILE){
	open my $ID, '<',$IDFILE;
	chomp;
	s/\r$//;
	s/\s+$//;
	next if (/^$/);
	next if (/^\s+$/);
	#s/\s+/_/g;
	my $id=(split /\s+/, $_)[0];
	$target{$id}=1;
}

$/='>';
while(<$FAS>){
	chomp;
	next if(/^$/);
	next if(/^\s+$/);
	my $entry=(split /\n/,$_)[0];
	$entry=~s/\s+/_/g;
	$entry=~s/:+/_/g;
	$entry=~s/\W+/_/g;
	my $outfile= $mode==0 ? $entry : substr($entry,0,$mode);
	open my $OUT,">>",$outfile.".fas";
	if(! defined $OUT){
		warn(" failed to creat file for this entry: $entry\n");
		next;
	}
	next if (defined $IDFILE && ! defined $target{$entry});
	print $OUT '>';
	print $OUT $_;
	close $OUT;
}
```

```
close $FAS;

#!/usr/bin/env perl

use strict;
use warnings;
use Getopt::Long;

my $opt={};
GetOptions ($opt, 'id=s', 'fasta=s','help|h!');

if(defined $opt->{help} || !defined ($opt->{id}) || !defined $opt->{fasta}){
	print STDERR "usage: perl $0 --id id.txt --fasta seq.fasta\n";
	print STDERR "      options:\n";
	print STDERR "            --id          seq id to be removed\n";
	print STDERR "            --fasta    fasta file to be processed\n";
	print STDERR "            --help      show this message\n";
	exit;
}

open my $ID, '<', $opt->{id} or die "$opt->{id}: $!\n";
open my $FA, '<', $opt->{fasta} or die "$opt->{fasta}: $!\n";

my %id=();
while(<$ID>){
	next if (/^#/);
	chomp;
	s/\s+$//;
	next if (/^$/);
	$id{$_}=1;
}
close $ID;

$/='>';
while(<$FA>){
	chomp;
	s/\>$//;
	next if(/^\s$/);
	next if(/^$/);
	my $header=(split /\n/, $_)[0];
	$header=~s/\s+$//;
	next if(defined $id{$header});
	print ">$_";
}
```

```
close $FA;

use strict;
use warnings;
use Getopt::Long;

my %opts;
    GetOptions ("Category!" => \$opts{Category},
                        "Subcategory!"      => \$opts{Subcategory},
                        "Subsystem!"    => \$opts{Subsystem},
                        "Role!" =>\$opts{Role},
                        "help!"=> \$opts{help} )
    or die("Error in command line arguments\n");

if (defined $opts{Category} || defined $opts{Subcategory} || defined $opts{Role}){
        if(scalar(@ARGV)>0){
                my $count={};
                my @files=();
                foreach my $file (@ARGV){

                        open my $fh,'<', $file;
                        if(!defined $fh){
                                warn "$file: $@\n";
                                next;
                        }
                        my $filter=";
                        while(<$fh>){
                                chomp;
                                next if(/^$/);
                                next if(/^\s+$/);
                                next if (/^Category\tSubcategory\tSubsystem/);
                                my ($Category, $Subcategory, $Subsystem,$Role,$Features)=split /\t/;
                                $Category=~s/\s+$//; $Category=~s/^\s+//;
                                $Subcategory=~s/\s+$//; $Subcategory=~s/^\s+//;
                                $Subsystem=~s/\s+$//; $Subsystem=~s/^\s+//;
                                $Role=~s/\s+$//;    $Role=~s/^\s+//;
                                $Features=~s/\s+$//;    $Features=~s/^\s+//;

                                # next if (defined $opts{Category} && ! grep( /$Category/,
@{$opts{Category}} ));
                                # next if (defined $opts{Subcategory} && ! grep( /$Subcategory/,
@{$opts{Subcategory}} ));
                                # next if (defined $opts{Subsystem} && ! grep( /$Subsystem/,
@{$opts{Subsystem}} ));
```

```
                # next if (defined $opts{Role} && ! grep( /$Role/, @{$opts{Role}} ));
                $filter=$filter."|".$Category if (defined $opts{Category});
                $filter=$filter."|".$Subcategory if (defined $opts{Subcategory});
                $filter=$filter."|".$Subsystem if (defined $opts{Subsystem});
                $filter=$filter."|".$Role if (defined $opts{Role});
                my @genes=split /,/,$Features;
                $count->{$filter}->{$file}+=scalar(@genes);
                $filter='';
            }
            push @files, $file;
            # print "$file\t$total";
            # if(defined $opts{Category}){
                # print "\t\"";
                # if (scalar(@{$opts{Category}})>1) {print join '|', @{$opts{Category}};}
                # else {print $opts{Category}->[0];}
                # print "\"";
            # }
            # if(defined $opts{Subcategory}){
                # print "\t\"";
                # if (scalar(@{$opts{Subcategory}})>1) {print join '|',
@{$opts{Subcategory}};}
                # else {print $opts{Subcategory}->[0];}
                # print "\"";
            # }
            # if(defined $opts{Subsystem}){
                # print "\t\"";
                # if (scalar(@{$opts{Subsystem}})>1) {print join '|', @{$opts{Subsystem}};}
                # else {print $opts{Subsystem}->[0];}
                # print "\"";
            # }
            # if(defined $opts{Role}){
                # print "\t\"";
                # if (scalar(@{$opts{Role}})>1) {print join '|', @{$opts{Role}};}
                # else {print $opts{Role}->[0];}
                # print "\"";
            # }
            # print "\n";
        }
        print "group";
        print "\t\"$_\"" foreach @files;
        print "\n";
        foreach my $FILTER(keys %{$count}){
            my $FILTER2=$FILTER;
            $FILTER2=~s/^\|//;
```

```
                print "\"$FILTER2\"";
                foreach my $FILE (@files){
                    print "\t";
                    print defined ($count->{$FILTER}->{$FILE} ) ? $count->{$FILTER}->{$FILE} :0;

                }
                print "\n";
            }
        }
        else {
            die "    at least one file to be specified\n";
        }

}
else {
    warn "\n    At least one of Category, Subcategory or Role must be provided for selecting genes\n\n";
    &help;
}

sub help{
    warn " usage: perl $0 [optiions]\n";
    warn "      options:\n";
    warn "            --Category           count genes number by Category\n";
    warn "            --Subcategory      count genes number by Subcategory\n";
    warn "            --Role                   count genes number by Role\n";
    warn "            --help                     show this message\n";
    exit(0);
}
```
